# Vasoplegic syndrome in patients undergoing heart transplantation

**DOI:** 10.3389/fsurg.2023.1114438

**Published:** 2023-02-13

**Authors:** Tong-xin Qin, Yun-tai Yao, the Evidence in Cardiovascular Anesthesia(EICA) Group

**Affiliations:** ^1^ Department of Anesthesiology, Shanxian Central Hospital, Heze, China; ^2^ Department of Anesthesiology, Fuwai Hospital, National Center for Cardiovascular Diseases, Chinese Academy of Medical Sciences, Peking Union Medical College, Beijing, China

**Keywords:** vasoplegic syndrome, heart transplant, risk factor, onset time, treatment

## Abstract

**Objectives:**

To summarize the risk factors, onset time, and treatment of vasoplegic syndrome in patients undergoing heart transplantation.

**Methods:**

The PubMed, OVID, CNKI, VIP, and WANFANG databases were searched using the terms “vasoplegic syndrome,” “vasoplegia,” “vasodilatory shock,” and “heart transplant*,” to identify eligible studies. Data on patient characteristics, vasoplegic syndrome manifestation, perioperative management, and clinical outcomes were extracted and analyzed.

**Results:**

Nine studies enrolling 12 patients (aged from 7 to 69 years) were included. Nine (75%) patients had nonischemic cardiomyopathy, and three (25%) patients had ischemic cardiomyopathy. The onset time of vasoplegic syndrome varied from intraoperatively to 2 weeks postoperatively. Nine (75%) patients developed various complications. All patients were insensitive to vasoactive agents.

**Conclusions:**

Vasoplegic syndrome can occur at any time during the perioperative period of heart tranplantation, especially after the discontinuation of bypass. Methylene blue, angiotensin II, ascorbic acid, and hydroxocobalamin have been used to treat refractory vasoplegic syndrome.

## Introduction

Vasoplegic syndrome (VS) is a common life-threatening complication characterized by severe and persistent systemic arterial hypotension (mean arterial pressure, <50 mmHg), normal or slightly increased cardiac output (cardiac index, >2.5 L/min/m^2^), low systemic vascular resistance (SVR, <800 dyne/s/cm^5^), and insensitivity to appropriate fluid resuscitation and high-dose vasopressors (1). VS occurs in up to 34.8% of patients who undergo heart transplantation (HTX) (2). The incidence of VS is higher in patients who underwent HTX compared to other forms of cardiac surgery, e.g., off-pump coronary artery bypass graft (CABG) (2.8%) (3), on-pump CABG (6.9%–26%) (3, 4), and aortic valve replacement (AVR) (20%) (5). Earlier research (6) showed that the incidence of VS is as high as 45% in patients with a ventricular assist device (VAD) at the time of HTX. Chemmalakuzhy et al. (7) observed increased risk for early mortality among HTX recipients with VS, with a 30-day mortality rate of 33%. This study aimed to summarize the risk factors, onset time, and treatment of VS in patients undergoing HTX.

## Materials and methods

### Search strategy

Relevant case reports were searched using the PubMed and OVID electronic databases from inception until January 14, 2022. Chinese literatures from the CNKI, VIP, and WANFANG databases were also searched. Different combinations of terms that included “vasoplegic syndrome,” “vasoplegia,” “vasodilatory shock,” and “heart transplant*” were used in the search strategy. All relevant case reports were included. The exclusion criteria were as follows: (a) non-English and non-Chinese studies; (b) studies based on animal models; and (c) duplicate publications. Each author independently read the titles and abstracts of all the identified reports for eligibility, excluding ineligible reports. The eligibility of the remaining reports for final inclusion was determined by examining the full-text versions of the publications.

### Data abstraction

Data of interest from the included case reports were abstracted and tabulated by each author independently: (a) author, year, and journal of publication; (b) total number of patients, age, sex, medical history, number of thoracotomy surgeries, bleeding and coagulopathy or not, postoperative transesophageal echocardiography, treatment of ventricular dysfunction (VD), and complications; (c) onset time, clinical manifestation, and treatment of VS. Disagreements were resolved by discussion between both authors during the process of data abstraction.

## Results

**Figure 1:**
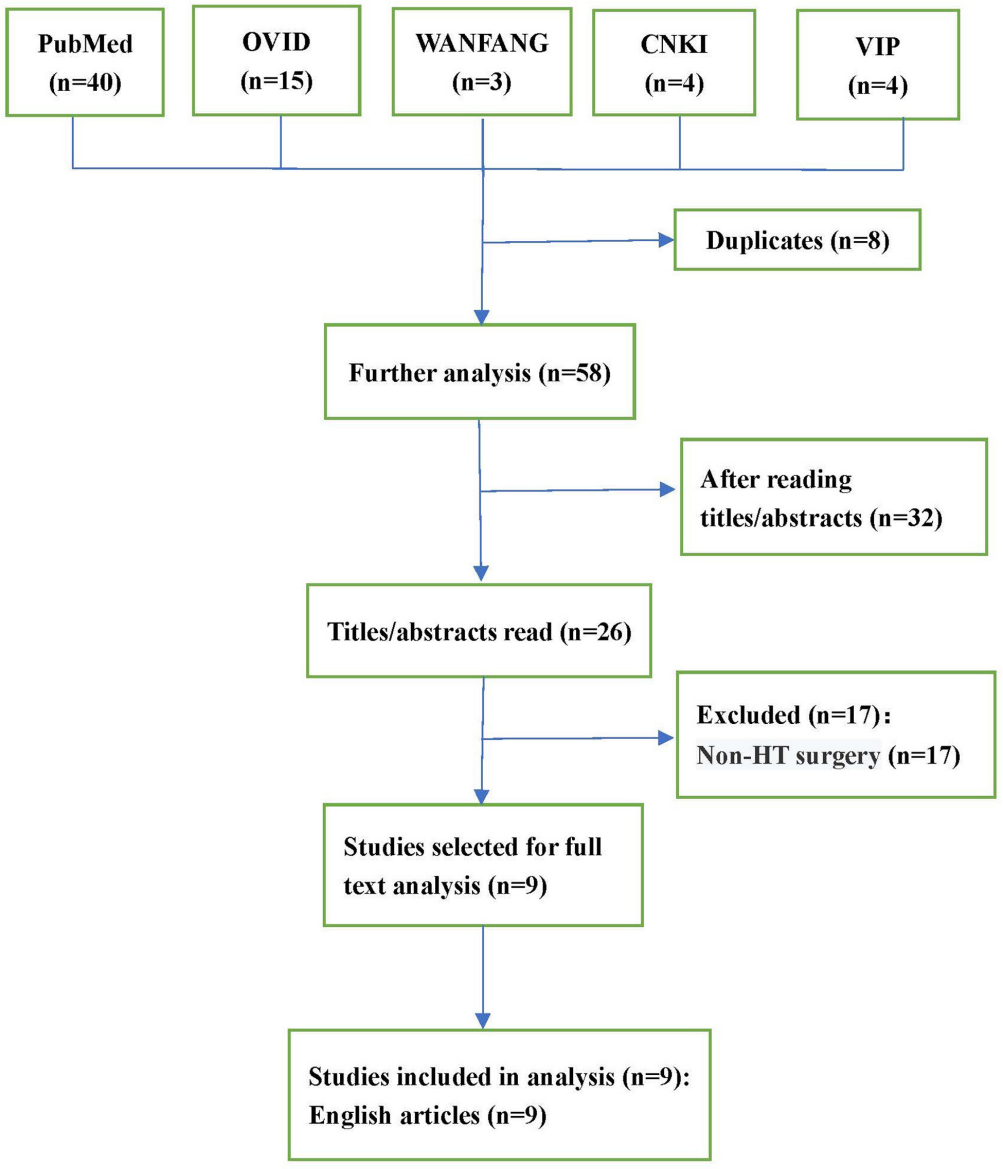
Flow diagram of study selection.

As depicted in the flowchart (Figure 1), the database search identified 26 potentially eligible studies. Nine case reports (8–16) describing 12 patients in total were deemed eligible and included. All case reports were written in English. A descriptive analysis of these cases is presented in Table 1. The 12 patients were aged 7–69 years, and included 9 males (75%) and 3 females (25%). Nine (75%) patients (8–11, 13–16) had nonischemic cardiomyopathy, and three (25%) patients (12, 13) had ischemic cardiomyopathy. Eight (66%) patients (10, 11, 13, 15, 16) had undergone preoperative thoracotomy, such as CABG, AVR, VAD, and Fontan operations. Five (63%) patients experienced intraoperative bleeding and coagulopathy intraoperatively due to the formation of dense adhesions between the mediastinum and pericardium. Six (50%) patients (8, 11–14, 16) used a variety of drugs before surgery, including angiotensin-converting enzyme inhibitors (ACEI), angiotensin II (ANG-II) receptor blockers (ARB), diuretics, β-blockers, and milrinone. After the discontinuation of bypass, eight (66%) patients (9, 12–14, 16) developed ventricular dysfunction, and were treated with milrinone, dobutamine, epinephrine, or norepinephrine. Other treatments include the inhalation of nitric oxide or epoprostenol, restarting cardiopulmonary bypass (CPB), and intra-aortic balloon counter-pulsation or extracorporeal membrane oxygenator.

**Table 1:** General condition and characteristics of patients experienced vasoplegic syndrome undergoing heart transplantation.

Cases	Age/Sex	Medical history	Thoracotomy #	Bleeding and coagulopathy	Post-TEE	Treatment of VD	VS		Complications
							Clinical manifestation	Treatment	
Kofidis 2001 (8)	55/M	ICM, HF, ACEI, Diuretics, Amiodarone, BB	1	NO	–	–	Persistent hypotensive, Increased requirement of NE, SVR < 500 dyne/s/cm^5^	MB 2 mg/kg IV over 30 minutes	Renal dysfunction
Wieruszewski 2019 (9)	47/M	NICM, HF, Obesity, CKD, SA septicemia	1	NO	RVD	Milrinone, EPI, Isoproterenol, Inhaled NO, NE	Persistent hypotensive, Increased requirement of NE	AA 1.5 g IV every 6 hours, ANG-II initiated at 20 ng/kg/min	–
Wieruszewski 2019 (10)	34/F	ICM, CHD, Fontan operation	2	NO	–	–	Persistent hypotensive, Increased requirement of NE and VP	ANG-II initiated at 20 ng/kg/min	–
Zundel 2015 (11)	53/F	ICM, LVAD, Obesity, Amiodarone	2	NO	–	–	Persistent hypotensive, Increased requirement of phenylephrine and VP, MAP < 50 mmHg	MB IV (Several times), Droxidopa 200 mg oral followed by 400 mg oral 3 times per day	Renal dysfunction, Respiratory insufficiency
Almufleh 2017 (12)	61/M	NICM, Smoke, BB, Sacubitril/valsartan	1	NO	BVD	Restart CPB, NE, Milrinone, EPI, IABP	Persistent hypotensive, Increased requirement of NE, SVR < 500 dyne/s/cm^5^, MAP < 50 mmHg	MB 2 mg/kg IV	Renal dysfunction
Cutler 2020 (13)	62/M	NICM, LVAD, RA, Serratia marcescens infection	2	YES	BVD	Dobutamine, Milrinone, Inhaled epoprostenol, IABP	Persistent hypotensive, Increased requirement of NE and VP, MAP < 50 mmHg	ANG-II 10–60 ng/kg/min, MB 2 mg/kg IV	Renal dysfunction, ION, SDH, Delay closed chest
Cutler 2020 (13)	61/M	NICM, LVAD, CABG, CKD	3	YES	BVD	Milrinone, EPI, Inhaled epoprostenol, IABP	Persistent hypotensive, Increased requirement of NE and VP, MAP < 50 mmHg	ANG-II 2.5–60 ng/kg/min, MB 1.5 mg/kg IV, Hydroxocobalamin 5 g IV	Renal dysfunction, Respiratory failure, Delay closed chest
Cutler 2020 (13)	69/M	NICM, CABG twice, DM, OSA, Milrinone	3	YES	BVD	Milrinone, EPI, Inhaled epoprostenol, IABP	Persistent hypotensive, Increased requirement of NE, MAP < 50 mmHg, SVR < 800 dyne/s/cm^5^	Hydroxocobalamin 5 g IV, ANG-II 5–30 ng/kg/min	Renal dysfunction, Liver injury, TP
Cutler 2020 (13)	60/M	NICM, LVAD, Obesity, LA	3	YES	BVD	Milrinone, EPI, Inhaled epoprostenol, IABP	Persistent hypotensive, Increased requirement of NE, SVR < 500 dyne/s/cm^5^	ANG-II 5–80 ng/kg/min	Renal dysfunction, TP, Agitated delirium
Bozzetti 2007 (14)	30/M	NICM, HF, ACEI, Diuretics, BB, Digoxin	1	NO	BVD	Milrinone, NE, Inhaled NO, ECMO	Persistent hypotensive, Increased requirement of NE, MAP < 50 mmHg	MB 2 mg/kg IV	–
Grubb 2012 (15)	60/M	NICM, LVAD, HF, Obesity, AVR, DM, HTN, CKD, Infections, Gout, Depression	3	NO	–	–	Persistent hypotensive, Increased requirement of phenylephrine, MAP < 50 mmHg	MB 1 mg/kg IV then 0.5 mg/kg/h	Serotonin syndrome
Lee 2016 (16)	7/F	NICM, LVAD, RVAD, Warfarin	3	YES	BVD	Milrinone, EPI, Inhaled NO	Persistent hypotensive, Increased requirement of VP, Insensitive to fluid resuscitation	MB 1.5 mg/kg IV	Delay closed chest

*M, male; F, female; NICM, nonischemic cardiomyopathy; ICM, ischemic cardiomyopathy; HF, heart failure; ACEI, angiotensin-converting enzyme inhibitors; HCM, hypertrophic cardiomyopathy; CKD, chronic kidney disease; SA, staphylococcus aureus; CHD, congenital heart disease; LVAD, left ventricular assist devices; RVAD, right ventricular assist devices; BB, β-blockers; CABG, coronary artery bypass grafting; HTN, hypertension; RA, rheumatoid arthritis; DM, diabetes mellitus; OSA, obstructive sleep apnea; LA, lupus anticoagulant; AVR, aortic valve replacement; TEE, transesophageal echocardiography; VD, ventricular dysfunction; VS, vasoplegic syndrome; RVD, right ventricular dysfunction; BVD, biventricular dysfunction; NE, norepinephrine; EPI, epinephrine; CPB, cardiopulmonary bypass; IABP, intra-aortic balloon counter-pulsation; NO, nitric oxide; ECMO, extracorporeal membrane oxygenator; VP, vasopressin; MAP, mean arterial pressure; SVR, systemic vascular resistance; AA, ascorbic acid; ANG-II, angiotensin II; IV, injection of vein; MB, methylene blue; ION, ischemic optic neuropathy; SDH, subdural hematoma; TP, thrombocytopenia.*

Of the 12 patients, 9 (75%) developed various complications, with 7 (58%) patients having developed some degree of renal dysfunction, respiratory insufficiency, ischemic optic neuropathy, subdural hematoma, thrombocytopenia, liver injury, agitated delirium, serotonin syndrome, or delayed chest closure. Most patients were discharged, but one patient (11) died of multiple organ failure.

**Figure 2:**
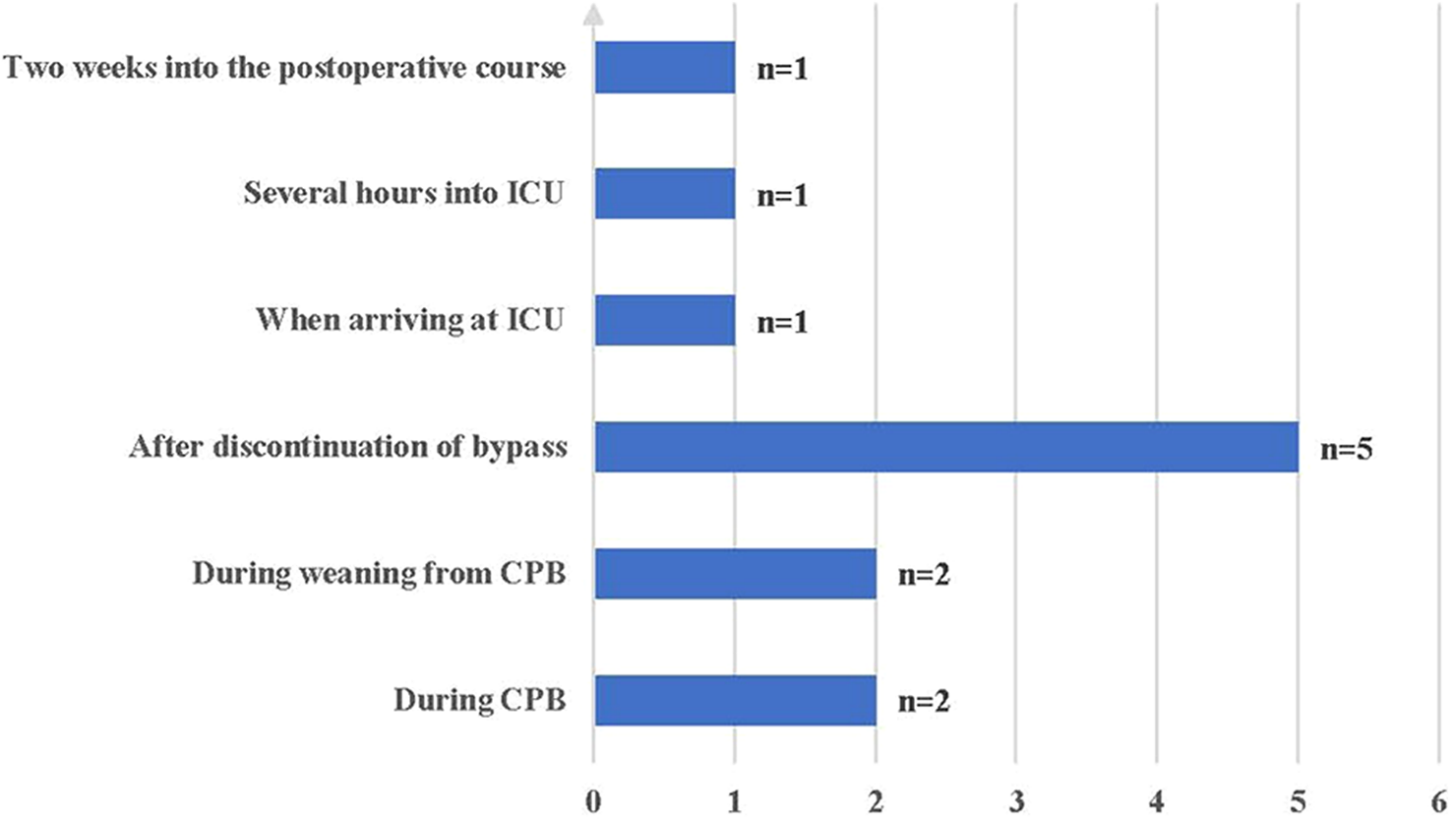
Onset time of vasoplegic syndrome. ICU, intensive care unit; CPB, cardiopulmonary bypass.

The time to onset of VS ranged from during CPB to 2 weeks postoperatively; nine (75%) patients experienced VS intraoperatively, and three (25%) patients experienced VS postoperatively (Figure 2). All patients were insensitive to vasoactive agents, developed persistent hypotension, and were subsequently administered methylene blue (MB), hydroxocobalamin, ascorbic acid (AA), and ANG-II.

## Discussion

Several risk factors for VS have been identified, including ACEI, β-blockers, calcium channel blockers, heparin, amiodarone, diabetes mellitus, prolonged CPB, congestive heart failure, and left ventricular ejection fraction <35% (17, 18). The preoperative use of VAD in adults is an independent risk factor for VS (6). In this study, six (50%) (11, 13, 15, 16) patients had used LVAD before surgery. Of the 12 patients, 8 patients (10, 11, 13, 15, 16) had undergone previous thoracotomy. This easily led to dense adhesions between the mediastinum and pericardium, resulting in severe bleeding and coagulation disorders, requiring a large number of blood products and factor replacement. Administration of blood products activates pro-inflammatory mediators during surgery (18). Packed red blood cells, fresh frozen plasma, and platelet transfusion increase the prevalence of VS (19). In addition, packed red blood cell transfusion exhibited a dose-dependent increase in the development of VS with each packed red blood cell unit transfused (19).

Milrinone is a powerful inotropic agent commonly used for right ventricular dysfunction, and may exacerbate systemic vasoplegia (20). Of the 12 patients, 8 (9, 12–14, 16) used milrinone pre- or intraoperatively. A meta-analysis (21) revealed that 38% of patients with New York Heart Association class III heart failure symptoms and 42% of those with class IV symptoms experienced depression. Depression not only increases the incidence of hypertension, coronary heart disease, and diabetes, but also causes chronic inflammation (22, 23). The mechanism of VS is largely unknown, and study results suggest that VS is correlated with the release of cytokines, such as tumor necrosis factor (TNF) and interleukin-1, which increase nitric oxide (NO) production, resulting in marked relaxation of the vascular smooth muscles (24). Therefore, the chronic inflammatory state of patients before surgery may be a risk factor for VS. Other chronic inflammation diseases include obesity, obstructive sleep apnea, chronic kidney disease, and smoke (25–28). Eight (67%) patients (9, 11–13, 15) had at least one of these medical histories. The risk factors for VS in the patients undergoing HTX are summarized in Table 2.

**Table 2:** Risk factors of VS in patients undergoing HTX.

**Preoperative:** ACEI ARB β-blockers CCB Heparin Amiodarone Milrinone Diabetes mellitus Hypertension CHF LVEF<35% VAD Thoracotomy surgery	**Intraoperative:** Milrinone Prolonged CPB Blood transfusion: Packed red blood cells Fresh frozen plasma Platelet

*ACEI, angiotensin-converting enzyme inhibitors; ARB, angiotensin receptor blocker; CCB, calcium channel blockers; CHF, congestive heart failure; LVEF, left ventricular ejection fraction; VAD, ventricular assist device; CPB, cardiopulmonary bypass; IABP, intra-aortic balloon counter-pulsation; ECMO, extracorporeal membrane oxygenator.*

Of the 12 patients, 9 (75%) experienced VS intraoperatively, including 4 patients before weaning from CPB and five after discontinuation of CPB. The other three patients had VS after arriving at the intensive care unit, and one developed VS 2 weeks post- operatively. Septic shock is considered more likely than VS 2 weeks after surgery. Therefore, the possibility of infection must be ruled out, especially infections of the chest, abdomen, genitourinary system, and bloodstream, which account for >80% of sepsis cases (29–31).

**Figure 3:**
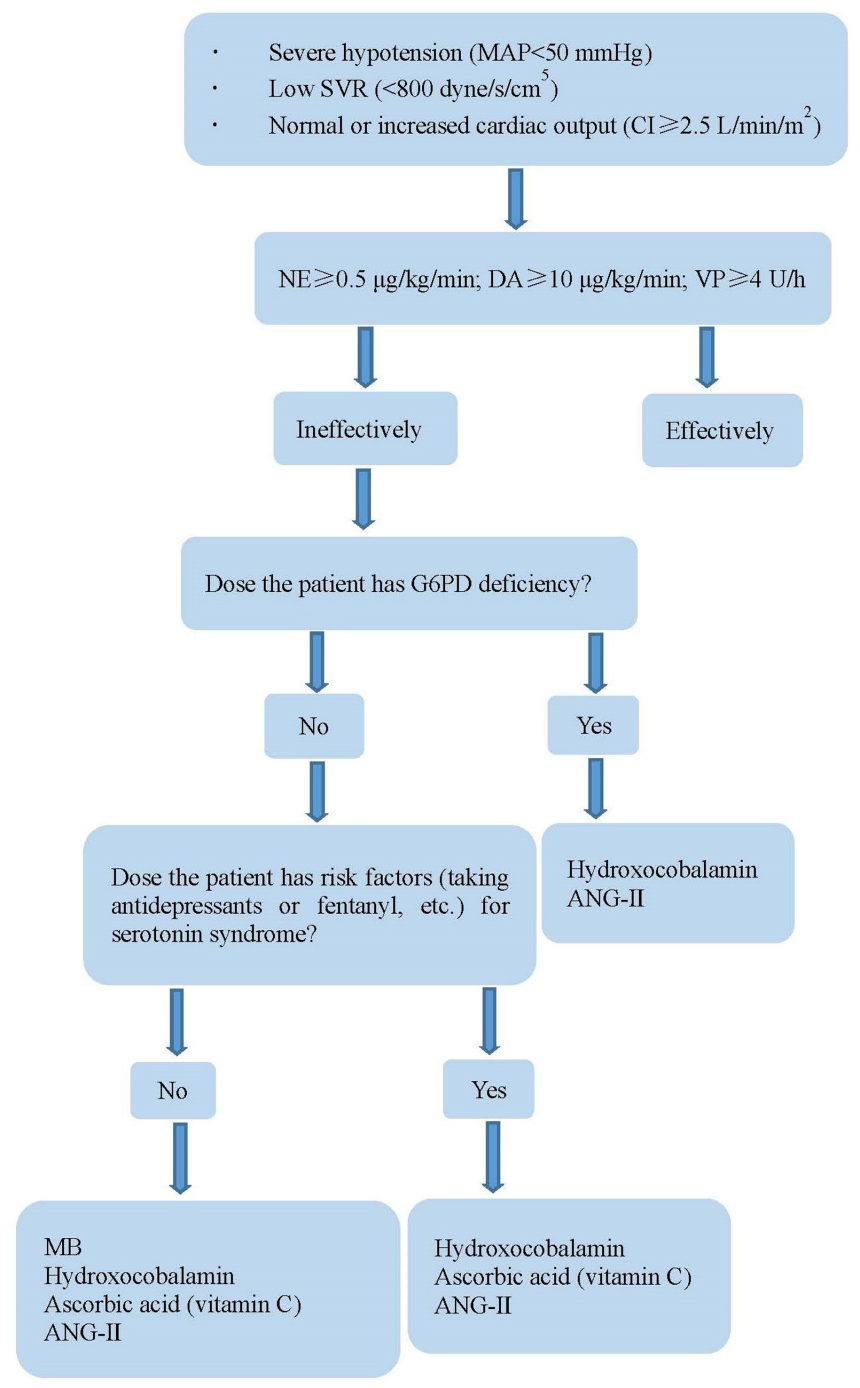
Treatment of vasoplegia during perioperative period of HTX. MAP, mean arterial pressure; NE, norepinephrine; DA, dopamine; VP, vasopressin; SVR, systemic vascular resistance; CI, cardiac index; G6PD, glucose-6-phosphate dehydrogenase; MB, methylene blue; AA, ascorbic acid; ANG-II, angiotensin II.

When VS occurs, catecholamines and vasopressin should be used at first. However, high-dose catecholamines may lead to tissue hypoperfusion and myocardial ischemia. Furthermore, prolonged hypotension may have adverse consequences, such as gradual deterioration of ventricular function and decreased urine output. At present, four drugs are used to treat refractory VS (Table 3). MB and hydroxocobalamin increase SVR by inhibiting NO synthase and reducing NO production, inhibiting the activation of soluble guanylyl cyclase, and binding to NO directly (32–35). Of the 12 patients, four were treated with at least two of these drugs. The combination of MB and hydroxocobalamin may be more beneficial than that of MB alone (36, 37). One study (38) found that MB reduced the duration of VS and mortality. However, a potentially lethal complication of MB is serotonin syndrome, especially in patients taking serotonergic antidepressants. Fentanyl is the most commonly used narcotic analgesics, which reduces serotonin reabsorption; therefore, it should be used cautiously when fentanyl was used during surgery. Hydroxocobalamin, an injectable form of vitamin B12, interferes with dialysis treatment owing to an alarm of blood leak, which can be overcome by continuous renal replacement therapy (39). AA is an essential cofactor for the endogenous biosynthesis of catecholamines, which cannot be synthesized by humans, and the concentration of AA in patients undergoing cardiac surgery after CPB is low (40–42). One study (43) found that the utilization of vasopressors was reduced when high-dose AA was administered for the treatment of VS after CPB. However, it should be noted that MB and AA cannot be used in patients with glucose-6-phosphate dehydrogenase deficiency to avoid hemolytic anemia. Prolonged exposure to CPB impairs the pulmonary capillary endothelium, thereby limiting the activity of angiotensin-converting enzyme (44). ANG- II acts directly on blood vessel walls, resulting in vasoconstriction, increased mean arterial pressure antidiuretic hormone secretion, adrenal cortex stimulation, and increased water reabsorption (44, 45). The adverse effects of ANG- II include thromboembolic events, hypoperfusion from vasoconstrictive actions, and increased pulmonary vascular resistance (46, 47). VS treatment of during the perioperative period of HTX is shown in Figure 3.

**Table 3:** Mechanisms and usages of the four drugs.

Drugs	Mechanisms	Usages
MB	Inhibit nitric oxide synthase Inhibit soluble guanylyl cyclase Directly binding NO	Adult 2 mg/kg IV over 30 minutes Child 1.5 mg/kg IV over 30 minutes
Hydroxocobalamin	Inhibit nitric oxide synthase Inhibit soluble guanylyl cyclase Directly binding NO Directly binding sulfide	5 g IV over 15 minutes
AA	Cofactor for endogenous biosynthesis of catecholamines Diminish induction of nitric oxide synthase Increase the sensitivity to catecholamines through reduction of adrenergic receptors to a basic state	1,500 mg IV per 6 hours
ANG-II	Constrict the blood vessels directly	Started at 20 ng/kg/min and then titrated to 40 ng/kg/min

*MB, methylene blue; AA, ascorbic acid; ANG-II, angiotensin II; NO, nitric oxide; IV, injection of vein.*

In addition to the abovementioned four drugs, induced mild hypothermia may be a useful treatment for VS. Earlier studies (48) showed that hypothermia decreases the release of cytokines. Furthermore, mild hypothermia effectively restored SVR and blood pressure within 4 h without adverse effects on pulmonary pressure (49), and improved the response to epinephrine (50) and norepinephrine (51). Therefore, it may be an excellent prevention and treatment method for VS by avoiding active rewarming after the operation and letting the patient gradually and spontaneously reach normothermia or maintain a 33°C–35°C corporeal temperature for the first 24 h after HTX. However, hypothermia can induce problems, such as cardiac arrhythmia and coagulopathy. Further research is necessary to determine the safety of mild hypothermia for the treatment of VS.

In-hospital mortality was more than 2.5-fold higher in patients with (25%) than in patients without VS (52). Therefore, the prevention of VS is crucial for patients undergoing HTX. Ozal et al. (4) reported that those who received preoperative MB had significantly higher postoperative SVR and MAP, and a significantly shorter mean length of stay in intensive care units. A randomized, double-blind, controlled trial showed that tranexamic acid attenuates the development of VS after CPB by blocking fibrinolysis (53). Further research should prioritize the mechanism and prevention measures for VS in patients undergoing HTX.

In summary, several risk factors for VS exist in patients undergoing HTX, including the chronic inflammatory exhibited by some patients before surgery. VS can occur at any time during the perioperative period in patients who underwent HTX, especially after the discontinuation of bypass. MB, ANG- II, hydroxocobalamin, and AA have been used to treat refractory VS.

## Data Availability

The original contributions presented in the study are included in the article/Supplementary Material, further inquiries can be directed to the corresponding author.
